# Mitochondrial protein synthesis: Figuring the fundamentals, complexities and complications, of mammalian mitochondrial translation

**DOI:** 10.1016/j.febslet.2014.05.054

**Published:** 2014-08-01

**Authors:** Robert N. Lightowlers, Agata Rozanska, Zofia M. Chrzanowska-Lightowlers

**Affiliations:** a The Wellcome Trust Centre for Mitochondrial Research, Institute for Cell and Molecular Biosciences, Newcastle University, The Medical School, Framlington Place, Newcastle upon Tyne NE2 4HH, UK; b The Wellcome Trust Centre for Mitochondrial Research, Institute of Neuroscience, Newcastle University, The Medical School, Framlington Place, Newcastle upon Tyne NE2 4HH, UK

**Keywords:** Mitochondria, Ribosomes, Translation, Gene expression, RNA

## Abstract

Mitochondrial protein synthesis is essential for all mammals, being responsible for providing key components of the oxidative phosphorylation complexes. Although only thirteen different polypeptides are made, the molecular details of this deceptively simple process remain incomplete. Central to this process is a non-canonical ribosome, the mitoribosome, which has evolved to address its unique mandate. In this review, we integrate the current understanding of the molecular aspects of mitochondrial translation with recent advances in structural biology. We identify numerous key questions that we will need to answer if we are to increase our knowledge of the molecular mechanisms underlying mitochondrial protein synthesis.

## 1 Introduction

It has been known for over 50 years that isolated rat liver mitochondria can incorporate radiolabelled amino acids into nascent polypeptides [1–4]. Although these first reports were challenged by researchers claiming that this incorporation was due to bacterial contamination of isolated mitochondrial preparations [5], by the late 1960s it had become well accepted that mammalian mitochondria were capable of intraorganellar synthesis of proteins [6,7]. We now know that the mitochondrial genome (mtDNA), which is housed in the mitochondrial matrix, contains the blueprint for thirteen polypeptides and all the RNA molecules believed to be necessary and sufficient for intramitochondrial protein synthesis [8]. All the other required components are imported from the cytosol. During this 50-year period, many factors have been identified that are critical for mitochondrial translation, but despite this we are still surprisingly unsure about many details underlying this process. This is due in major part to the lack of a faithful *in vitro* reconstituted system. Progress is further impeded by our inability to use standard molecular genetic manipulations, as there is no robust process for transfecting mammalian mitochondria [9,10]. In this short review, we mention important contributions to this field, but highlight fundamental questions that still remain.

## 2 What is so unusual about the mammalian mitochondrial ribosome?

Central to the process of mitochondrial protein synthesis is the mitochondrial ribosome, or mitoribosome. Pioneering work from O’Brien, Spremulli and others, showed that the bovine mitoribosome comprises 2 subunits of unequal size, a 28S small subunit (mt-SSU) and 39S large subunit (mt-LSU) [11]. Only one molecule of relatively short mtDNA-encoded ribosomal RNA could be identified in each subunit of the human mitoribosome – 12S rRNA in the small subunit (954 nt) and 16S rRNA in the large subunit (1559 nt) (however, see recent observations below) [8]. Intact mitoribosomes from a variety of mammalian sources were shown to be less dense (55S) than either their cytosolic (80S) or eubacterial (70S) counterparts and even differed from other organellar sources, such as *Saccharomyces*, *Neurospora*, *Tetrahymena* or *Xenopus* mitochondria [11–15] reviewed in [16]. This is largely due to a reversal in their protein to RNA ratio, changing from approximately 1:2 protein:RNA for eubacterial/eukaryotic cytosolic ribosomes to approximately 2:1 for the mammalian mitoribosome. The reduced ribosomal RNA species have not become shortened through stochastic loss of nucleotides but by selective excision of regions, including the anti-Shine–Dalgarno region, consistent with a corresponding lack of S–D sequence in mammalian mt-mRNAs. Although conservation of certain domains is clear, such as the sarcin–ricin loop and helix 45 of the SSU [17], there is little overall preservation of actual nucleotide sequence or even base composition [18]. Loss of part of the rRNA species would have been expected to reveal a number of spatial domains in a standard ribosomal structure. Intriguingly, some but not all of these domains have become occupied by a series of ‘newly acquired’ mitoribosome-specific proteins that have no apparent orthologues [17,19,20]. One consequence of these changes is a more porous structure, which is consistent with the original data indicating that a mitochondrial monosome had a low sedimentation coefficient of 55S [11,17].

Analysis of the many polypeptide constituents from a variety of mitoribosomal sources has been an iterative enterprise reflecting the constant technological improvement in detection methods [19,21–26]. Mass spectrometry of peptide fragments from the 39S large subunits of isolated bovine mitochondria identified 48 independent gene products. Many of these proteins could be assigned positions in an early and seminal cryo-EM structure from Agrawal and colleagues of the entire 55S mitoribosome at 13.5 A resolution [17,27]. Crystals of the mitochondrial ribosome have been elusive, but in their absence cryo-EM has continued to provide vital structural information, with improvements increasing the resolution to 12.1 Å of the bovine mt-LSU [27] and to 7 Å for the mt-SSU [28]. Recently, however, Greber and colleagues, using a combination of techniques, have produced a structure of the mt-LSU at 4.9 Å that is approaching the resolution achieved with crystallography. By subjecting porcine 39S mt-LSU preparations to chemical cross-linking followed by controlled proteolysis and MS analysis, contacts between numerous polypeptides have been unequivocally established. This information combined with the near-atomic resolution of the cryo-EM has both increased the number of mt-LSU assigned proteins to at least 51 members and identified the positions within the porcine mt-LSU of a number of these recently identified polypeptides [20]. Intriguingly, the structural analysis has also identified an RNA component that does not correspond to the 16S rRNA (see below). Currently there is no parallel study on the 28S mt-SSU, although previous mass spectrometry and increasingly sensitive analyses have revealed that it comprises at least 30 individual mitoribosomal proteins (MRPs) [18,23,24,29,30]. Cryo-EM has also very recently been used to generate high definition structural information on the yeast mitochondrial ribosome, but again it is the large subunit rather than the small that has been investigated. Single particle cryo-EM, using high-speed direct electron detectors, has been used to produce an almost complete model of this mt-LSU [31]. In this case there is no evidence of a 5S rRNA particle, consistent with the lack of the 5S RNA binding proteins L18 and L25 [31]. As a consequence of the loss of these elements, the central protuberance is significantly remodelled, with mitochondrial specific proteins occupying the vacated space. The yeast mt-LSU has 8 mitochondrial specific proteins that are common to both yeast and mammals, but a further 5 that so far are believed to be specific to yeast [31]. The accumulated data raises the question *– are these really yeast specific or are there still more components to the mammalian mitoribosomes that, as yet, have escaped detection?*

## 3 New versus old ribosomal proteins

As mentioned above, mitoribosomes have acquired a number of new protein components. This means that the MRPs can be divided into two groups, new and old and these are roughly equal in number The old group includes those with clear eubacterial orthologues, evidencing the bacterial origin of the mitochondria, which therefore follow a similar nomenclature (e.g. MRPL1 is the orthologue of RPL1). The second group of ‘new’ mitochondrial specific MRPs (reviewed in [18,31,32]) appears to be evolving more rapidly than cytosolic ribosomal proteins and have adopted functions that suggest they do not merely act as fillers to occupy the space generated by the reduced rRNAs [33–36]. Acquisition of these novel mitoribosomal proteins appears to be through gene duplication or through the requisition of non-ribosomal proteins that have become targeted to mitochondria, often bearing post-translational modifications (discussed in [37]). One clear example of such gene duplication in mammals results in the presence of MRPS18A, B and C [24]. The difference in function of these distinct isoforms has not yet been elucidated, but tissue specificity, or the formation of specialised ribosomes dedicated to the translation of subsets of mt-mRNAs, are potential explanations. The acquisition and adaptation of pre-existing proteins is a fascinating phenomenon. A case in point is that of MRPL39, originally termed MRPL5 [21,26]. A heart specific variant of this protein was identified, which displayed sequence similarity to the N-terminal domain of cytosolic threonyl-tRNA synthetase that had maintained its tRNA binding site [38]. Adaptive evolution presumably dispensed with the mid and C-terminal regions, leaving a mitoribosomal protein with a currently undefined function. *Has this substantial increase in the relative amount of protein only evolved to shield the rRNA from damaging reactive oxygen species as speculated by a number of groups, or are there other novel functions still waiting to be disclosed?*

In contrast to those novel proteins of unknown function that have been acquired by the mitoribosome, a number of other new MRPs have brought defined but unexpected functions to their new home. An example of such a protein is ICT1 (redefined as MRPL58 in [18]). The transcript encoding this protein was first reported in a cell culture model of colon carcinoma, where levels varied between differentiated and undifferentiated HT29-D4 cells [39]. Consequently, it became known as immature colon carcinoma transcript 1 (ICT1). This deceptive nomenclature delayed its recognition as an MRP. Characterisation of ICT1 later revealed that this protein exhibited peptidyl-tRNA hydrolase activity. Any uncontrolled ability to cleave the elongating peptide from the P-site tRNA is potentially lethal to the cell. It is therefore a somewhat surprising function to incorporate into the mature mitoribosome [40]. How this activity is restricted is part of an ongoing investigation in our laboratory. Other bifunctional MRPs with deceptive nomenclature exist, including the mt-SSU associated Programmed Cell Death Protein 9, PDCD9 (or MRPS30), a protein involved in apoptosis [24,41] and another mt-SSU component, Death Associated Protein 3, DAP3 (or MRPS29), also reported to be an apoptotic factor [42,43]. This mitochondrial specific protein brings a novel GTP-binding activity to the ribosome [35]. *Is it possible that further bifunctional proteins will be identified as important in the assembly, or as structural components of mammalian mitoribosomes?*

## 4 Are all mitoribosomes born equal?

It is often assumed that all ribosomes are constitutively active and are identical, irrespective of the different tissues, environmental cues or species of transcript to be translated. However, this is in contrast to the established concept of a ribosome filter, where translational control is exerted at the level of ribosome selection [44]. The application of this concept to mitoribosomes has only more recently been considered [45,46]. To customise a ribosome, potentially the protein composition or rRNA sequences could be varied. Examples of the latter occur in *Haloarcula marismortui*, which can synthesise highly divergent rRNA species [47] and in *Plasmodium falciparum* that expresses different rRNA species at different stages of its lifecycle [48], resulting in distinct ribosomal populations. No such variation is found in mammalian mt-rRNA, at least at the nucleotide sequence level, where the vast majority are identical, although variation in modification status cannot be ruled out. *If the rRNA does not vary, do variations in MRP composition occur?* The isoforms of MRPS18 and their potential in generating different 55S species has been described above. Omission, or potentially inclusion, of extra copies of MRPs could also vary the 55S particles. Variation in expression has been observed for a number of genes encoding MRPs, including MRPL11, MRPS23 [49] as well and MRPL28 [50,51]. In each case the resultant change in MRP composition was accompanied by significant changes in OXPHOS and oxygen consumption. Variation could also be introduced at the level of protein modification (see below). This suggests that different forms and compositions of mitoribosomes may exist, which by altering their translational activity or efficiency, can modulate cell metabolism. Further weight to this argument comes from recent plant mitochondria data. Kwasniak et al. report the artificial generation of polymorphic mitoribosome populations in *Arabidopsis* through siRNA-mediated depletion of a specific MRP. This single alteration in the levels of RPS10 altered the translation pattern within mitochondria [52] demonstrating that *Arabidopsis* mitoribosomes can regulate intra-organellar translation through transcript selectivity [52].

Calculations indicate that the nuclear encoded MRPs are evolving at a rate that matches that of the mt-rRNA [33], but is the evolution of multiple MRP isoforms evidence of different mitoribosomal populations? Perhaps similar proteins from a single family are interchanged under different physiological conditions, or are tissue specifically expressed. Only a limited number of investigations have prepared and analysed the amino acid sequences of MRP components from multiple tissues from the same organisms. Therefore it has not yet been possible to validate whether the alternative MRP transcripts are tissue specifically or developmentally expressed. Considering all this data, however, it is tempting to speculate that in the next few years there will be attempts to engineer mitoribosomes to exploit specialised functions.

## 5 Do mammalian mitoribosomes contain a 5S rRNA species?

A detailed discussion on the structures of the mitoribosome is beyond the scope of this mini-review but recent publications that we have already highlighted are highly recommended [18,20,31]. However, there is one issue that we find particularly intriguing. Perhaps the most striking difference between mammalian mitoribosomes and its other counterparts is the aforementioned reversal of RNA to protein ratio. This loss in ribosomal RNA extends beyond just the reduction in size of rRNAs, as an entire rRNA species appears to be missing. Until relatively recently, 5S rRNA had been documented as absent from 55S particles [17,18,22,32,45,53]. Further, the recent high resolution structure of the yeast mt-LSU also describes 5S rRNA and associated binding proteins to be absent [31]. One feasible explanation for the lack of 5S rRNA associated with the mt-LSU is the change in MRP composition. At least for yeast, results from the new cryo-EM support this unequivocally. The situation differs slightly in the mammalian counterpart. There are multiple proteins that make contact with the 5S rRNA species in the eubacterial ribosome. Two such proteins are L5 and L25; both are absent from mammalian mitoribosomes. A further protein, L31, which in the eubacterial LSU is found in close proximity to the 5S, lacks a mitochondrial equivalent [18,54,55]. In the absence of these proteins, it is not unreasonable to have concluded that the 5S rRNA has been lost as an integral component of the mt-LSU [17,18]. The original cryo-EM studies performed on bovine mitoribosomes revealed one molecule of 16S ribosomal RNA per subunit, with no additional density consistent with a smaller 5S or equivalent species, apparent [17]. Moreover, from the recent high resolution structural data of the porcine mt-LSU it can be seen that approximately 50% of the space that would have been occupied by a 5S rRNA molecule has been filled with MRPs [17].

Even now it is still unclear whether the mammalian mitochondrial ribosome incorporates more than just two molecules of rRNA. Whilst it may seem surprising in comparison to other ribosomal particles that the mammalian mitoribosome incorporates just one molecule of rRNA per subunit, it is consistent with the self sufficient eubacterial origins and the mammalian mtDNA encoding all the RNA species required for intramitochondrial protein synthesis. The synthesis of the 2 mt-rRNA species and all mt-tRNA, would preclude the necessity for importing any RNA species from the cytosol. Such an explanation, however, may be too facile and it would seem to be contradicted by a variety of other observations. First, there is evidence based on subcellular fractionation and *in vitro* studies that 5S rRNA is capable of being imported into mammalian mitochondria [56–59]. Second, PNPase in the intermembrane space of the organelle has been implicated in the import of physiologically relevant RNA species into mitochondria [60,61]. Third, affinity-captured mitoribosomes from cultured human cells has been claimed to contain substantial levels of 5S rRNA, approaching levels of 16S mt-rRNA but not 5.8S rRNA [56]. Finally, and most tantalisingly, the high resolution images of 39S porcine LSU reveal an RNA density that is separate from 16S rRNA. This rRNA density is found in close proximity to MRPL18, the orthologue of another eubacterial protein that associates with 5S RNA in the ribosome. It is in a similar position (at the central protuberance) to where the 5S might be expected, by analogy to the bacterial ribosome. Intriguingly, the density is too minimal to be a complete 5S species, although it does show similarity to the 5S rRNA domain β. In further contrast to 70S and 80S particles, this density does not contact the main body of the mt-LSU [20]. Indeed, part of the structural role that 5S rRNA plays in the eubacterial ribosome, appears to be compensated for by 2 mitochondrial specific proteins, MRPL38 and MRPL52. The lost structural contact with the main body of the mitoribosomal LSU is substituted by MRPL52, which is predicted to include a long α-helix. The latter in turn contacts MRPL38 at the top of the central protuberance, stabilising the whole structure [20].

Returning briefly to the issue of 5S rRNA import into mammalian mitochondria, this is an issue that has evoked much discussion. Mitochondria from many organisms require RNA species to be imported from the cytosol to support protein synthesis. This is particularly well recognised for transfer RNAs (reviewed in [62,63]), where 2 import systems have been characterised [63]. In contrast, PNPase has been implicated in augmenting the import of various other RNA species into human mitochondria, including the 5S rRNA. This surprising observation is supported in part by the main submitochondrial location of PNPase being between the two membranes. Although this protein is better known for its ability to degrade rather than transport RNA [64–66], Wang et al. performed a large number of *in vitro* and *in vivo* experiments in various species to support the claim, including the use of mutated PNPase to separate import and enzymatic functions. Interested readers are recommended to consult this work [60].

**Fig. 1:**
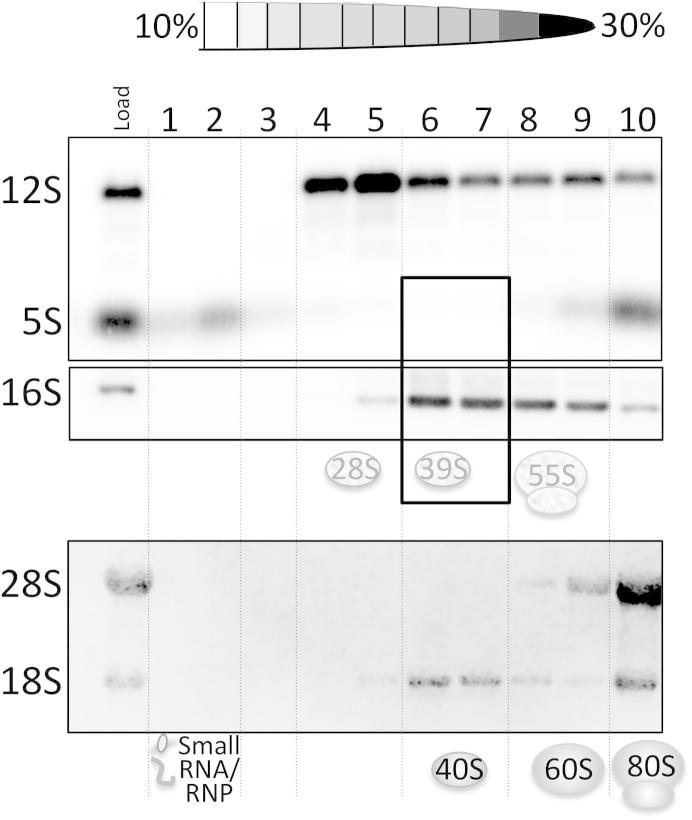
Intact *5S rRNA does not co-localise with the large subunit of mitochondrial mitoribosomes.* Cell lysates (850 μg), prepared from human HEK293 cells, were separated by isokinetic sucrose gradients [40]. RNA was isolated from each fraction (1 – 10) and the subsequent northern blot was interrogated to determine the distribution of ribosomal RNA species [79]. Positions of the cytosolic and mitochondrial small and large subunits are indicated underneath the panels. ‘Load’ represents RNA extracted from 85 μg cell lysate, equivalent to 10% of the lysate that was loaded onto the gradient. The position of the mt-LSU is boxed together with the corresponding fractions for the 5S rRNA panel.

*Does this additional density derive from 5S rRNA?* If so then it seems likely that this density will correspond to only a fragment of the entire 5S, necessitating a form of processing. *Is an alternative possibility that this RNA species is only transiently associated with the mt-LSU and is not present in the fully assembled 55S particle?* Although plentiful, the copies of cytosolic rRNAs vastly outnumber those of the mt-rRNAs. Moreover due to nuclear encoded but mitochondrially destined proteins that are co-translationally translocated across the outer mitochondrial membrane (OMM), it is difficult to purify mitochondria without cytosolic ribosomes anchored as co-contaminants. Protease shaving and RNase treatment with or without disruption of the OMM can significantly reduce but rarely eliminates all the 18S, 28S and 5S present in preparations, making qPCR analysis unreliable. So how might it be possible to convincingly discriminate between *bono fide* mitochondrial 5S and a contaminating population? We have performed a simple RNA isolation from fractions following isokinetic sucrose density gradients, which separates the mitoribosomal and cytosolic ribosomal subunits in total cell preparations. If 5S rRNA were present in fully assembled mt-LSU, it would be found in stoichiometric amounts relative to 16S rRNA species. A northern blot following such a fractionation of HEK293 cells is shown in Fig. 1. Probing for major rRNA components (5S, 12S, 16S, 18S and 28S) indicated that a small fraction of the 5S is incorporated into a low density particle as has been well described, previously [67], whilst the vast majority is associated with the 80S particle as indicated by the co-migration of 18S and 28S rRNA (Fig. 1). Clearly, there is no significant pool of 5S rRNA co-migrating with the16S rRNA, a marker of the mt-LSU, precluding any possibility that the 5S rRNA is present in stoichiometric amounts within the mt-LSU. Further, if the rRNA present is a processed shorter form of 5S, then it avoids detection by standard northern blot using the entire 5S species as probe. It is intriguing that a second RNA species is present in the porcine mt-LSU, but this simple fractionation experiment shows it is unlikely to be the 5S rRNA, unless it is weakly bound and/or subject to degradation.

## 6 Are there three available sites on the mammalian mitoribosome for mt-tRNA occupancy?

Another unresolved question is whether the mammalian mitoribosome contains an Exit or E-site for tRNAs following their translocation through the A- and P-sites, consistent with all other characterised ribosomes. Mears and colleagues evaluated the eubacterial contact points that tRNAs made with the ribosome in each of the A-, P- or E-sites and compared this with contact points that could be potentially retained in mammalian mitoribosomes [27]. In the A-site, 15 contact points were conserved and only 3 absent, whilst all 8 P-site contacts were conserved. Retention of these contact sites in the A- and P-site is understandable as accurate placement of the tRNA acceptor stem is vital for peptidyltransferase activity, necessary for elongation of the nascent peptide. This conservation was in strong contrast to the E-site where there was only 1 conserved but 11 absent contacts [27]. Loss of these contacts in the E-site has led to the suggestion that this feature may be weak or essentially absent in mammalian mitoribosomes [17,68]. This is consistent with the observation that unlike eubacterial ribosomes and indeed yeast mitoribosomes that retain tRNAs in the E-site on isolation, mammalian mitoribosomes tend to purify with the P-site rather than E-site occupied by tRNAs [17]. The explanation for this loss may lie in the unusual reversal of the RNA:protein ratio mentioned earlier.

## 7 How do nascent polypeptides leave the mitoribosome?

Not only are there changes in how the tRNA exits the mitoribosomes, it is clear that both the tunnel and exit site of the mitoribosome, through which the nascent proteins travel and emerge, have been remodelled compared to 70S and 80S particles [17,20]. One notable difference is that all the intraorganellar synthesised proteins are highly hydrophobic. They will, therefore, have different chaperone requirements compared to any soluble proteins that will be synthesised as part of the repertoire of cytosolic or eubacterial ribosomes. One interesting feature is a unique cavity, termed the polypeptide accessible site (PAS) that may form an alternative escape route for nascent polypeptides. This opening results from the loss of rRNA domains (equivalent to the 23S domains I and III) that would otherwise have lined part of the exit tunnel [17]. It has not been determined which newly synthesised proteins, if any, might use this site over the more conventional polypeptide exit site (PES). The PES is not entirely conventional as it too has new mito-specific MRPs in its architecture, forming a lid-like extra layer [17,20]. Presumably these mitochondrial specific proteins MRPL39, MRPL44 and MRPL45, which have been incorporated at this exit site [20], have evolved to facilitate accurate co-translational insertion of the nascent peptide into the IMM. It may be that different cohorts of chaperones flank these two sites, each specific to particular nascent polypeptides. *Is this an additional function that the new MRPs stationed at the exit proteins might serve?*

As described above, the mammalian mitoribosome has evolved to synthesise solely hydrophobic inner membrane proteins. The presence of the PAS and modifications to the PES are likely to facilitate the entry of these polypeptides directly into the inner membrane. Co-translational insertion of polypeptides will perforce anchor the mitoribosomes to the IMM but data initially from the Spremulli lab has shown that an interaction between mitoribosome and membrane can occur independent of translation [69]. Preparations of IMM and matrix were probed for the presence of mt-rRNA species and even following puromycin treatment of the IMM fraction to release nascent peptides, a large subset of mitoribosomes remained associated with the membrane [69]. The mito-specific MRPs have long been suggested anecdotally as potentially fulfilling this role but Greber et al. put forward a plausible model for MRPL45 acting as the anchor. The position of MRPL45 on the outer extremity of the exit site, together with a short helix that bears similarity to a putative membrane interacting segment of TIMM44, a component of the protein import machinery, make MRPL45 a likely candidate to keep the translating mitoribosome in close proximity to the IMM [20]. *If this protein does anchor the large subunit to the IMM does this mean that the complete mt-LSU is permanently attached?* In the work mentioned above, Liu and Spremulli determined that approximately half of the mt-rRNA was associated with the IMM. They did so by probing dot blots for mt-rRNA that had been extracted from either the matrix soluble or IMM fractions [69]. One interpretation could be that signal derived from the soluble 16S represents incomplete mt-LSU that is in the process of being assembled. This would leave some unanswered questions including - *How does the mt-SSU become associated with the mt-LSU? Does the mt-SSU only join the mt-LSU once mt-mRNA associates with the former? Are 55S particles only present at the IMM when they are actively translating or can 55S be free in the matrix?* It seems likely that new methods of detection will be needed to investigate these questions. Technological developments in imaging have improved dramatically since work on the mitoribosome began. Super-resolution microscopy in the form of PALM, STED, N-SIM, N-STORM (relative merits are reviewed in [70]) and others now give definition to ∼20 nm. These techniques have been used to resolve questions about intramitochondrial structures [71,72] but despite these advances in imaging, none of these methods are currently likely to be able to distinguish between complete monosomes versus individual subunits that are free in the matrix. Transmission EM can give better resolution but without suitable antibodies or reporters it will still limit detection of ribosomal subunits that are separate rather than part of the complete monosome. Correlative Light and Electron microscopy (CLEM) is not a new technique [73], but impressive improvements have been made since its instigation, with resolution now possible to ∼2 nm [74]. Genetic tags for use in CLEM have been designed by Roger Tsien, the doyen of GFP, and demonstrated to label mitochondria [75]. Perhaps this method of visualisation will be a way forward?

## 8 How are these molecular machines put together?

Biogenesis of cytosolic ribosomes requires over 170 proteins and a further 70+ small nucleolar RNAs. In contrast, eubacterial ribosomes, the ancestors of mitochondria, appear to require only 20 or so non-ribosomal proteins to coordinate assembly if one excludes the rRNA modifying enzymes [76]. Although considerably fewer than the trans-acting factors required for 80S assembly the number of factors identified as playing a role is likely to increase [77]. Remarkably this process of ribosome assembly is one that can be recapitulated *in vitro* (reviewed in [76,78]). The pathway of 55S biogenesis is currently very ill defined. *Will putting together mammalian mitoribosomes, which are so different in composition to their counterparts, require many or a minimal number of factors to effect assembly?* So far we know of only a few assembly factors, including those that chaperone or modify the mt-rRNAs [79,80], and those where their specific role is inferred, as their absence causes a disruption in mitoribosome biogenesis [81–84]. Ribosomal RNA modifications are limited and need further definition (reviewed in [85]). In some cases, methyltransferases have been implicated, although it has not been possible to demonstrate direct modification *in vitro*, possibly because the substrate, in the form of a partially assembled mitoribosomal subunit, is not available or that other co-activators may be required [86–88]. Mitoribosomal protein modification has also been identified. A thorough review by Koc and Koc has shown the overwhelming majority of MRPs to be either acetylated or phosphorylated [89]. However, the extensive proteomics approach did not determine the effects of these modifications on ribosome biogenesis [89]. With the exception of SIRT3 acting to acetylate MRPL10 [90], the proteins responsible for effecting the modifications, or indeed their functional significance remain largely undefined. Despite our increase in knowledge, many questions still remain*. When does the mt-rRNA become incorporated? Are there specific subcomplexes that are initially formed? Are the mt-LSU and mt-SSU assembled on the IMM surface or in the matrix? Are the rRNA modifications a pre-requisite for incorporation into the subunit or do they occur as part of a quality control process? Is there a quality control process that prevents aberrantly assembly subunits from associating to form a 55S particle?*

## 9 What is known about the natural process of mitochondrial translation?

Having assembled the mitoribosomal subunits, it is not yet fully understood how an mt-mRNA is loaded to facilitate translation. Various *in vitro* analyses by Spremulli and colleagues have investigated the association of mt-mRNAs with the small subunit and estimated what impact the structure of the 5′ termini of the transcripts might have on loading [91–94]. Thus far the data suggests that the initial step is the association of mt-mRNA and fmet-tRNA^met^ with the small subunit, assisted by initiation factors that have been characterised, again by the Spremulli group [95–101]. This is a GTP dependent process but the details and timing of these initiation events have yet to be confirmed. *In vitro* derived data confirmed a strong preference for the initiating AUG to be placed at the very 5′ terminus. As few as three nucleotides upstream of the start codon resulted in very inefficient initiation, strongly supporting the need for processing to have completely excised the transcripts from the primary polycistronic unit prior to translation [91]. The lack of 5′-UTRs longer than 3 nucleotides on the mt-mRNAs, with the exception of the internal start sites of *MTATP6* and *MTND4*, also means that transcripts could potentially act like leaderless bacterial mRNAs, which can join fully assembled ribosomes rather than via an initial SSU interaction [102–104]. In addition to confirming precisely how these transcripts associate and initiate translation, the intriguing question still remains – *how do the mitoribosomes find the internal AUG initiation codons in each of the two bicistronic transcripts?*

Following initiation, peptide elongation occurs through a basic mechanism that is similar to the eubacterial counterpart [97–102]. Termination and recycling also share numerous characteristics, although the unique mitochondrial genetic code produces some unusual anomalies. For example the original interpretation of the human mitochondrial genetic code suggested that AGA and AGG had been recoded from triplets encoding arginine, to STOP codons [8]. This led to the search for an omnipotent release factor that could recognise and terminate translation from 4 codons, UAA, UAG, AGA and AGG that were believed to be used by many mammalian species [105]. The original candidate, mtRF1, was found not to be able to facilitate translational release at any termination codon, but identification of a second family member, mtRF1a (or MTRF1L [106]), exhibiting a greater similarity to standard release factors, was able to promote peptide release from a UAA and UAG codon [107]. Further *in vivo* data has shown that on entry of the single AGA or AGG triplet to the mitoribosomal A-site, a -1 frame shifting event occurs, placing a standard UAG into the A-site, therefore allowing mtRF1a to effect polypeptide release for all thirteen human mt-encoded polypeptides [108]. The mitochondrial release factor family includes two other members, ICT1 (see above) and C12orf65, mutations in the latter are known to cause clinical manifestations common to mitochondrial disease [109–113]. Depletion or mutation of these factors causes defects in mt-translation but their specific function remains unknown [109,114,115]. Following termination, the mitoribosome and mt-mRNA need to dissociate to perform a new round of translation. The features of this process have been characterised and it is likely that research will contribute only minor refinements to our understanding [105,116,117].

Many questions remain in this arena too. Is there premature drop-off from abortive translation? What mechanisms recognise and eliminate the aberrant peptide and the rogue mt-mRNA? This review does not touch upon the processing, maturation or stability of the mt-transcripts or the process of turnover but these areas are also under intense investigation.

## 10 Is the mitoribosome part of a large mitochondrial ‘expression factory’?

Mitochondrial nucleoids were initially described as a complex containing mtDNA and various protein components, although their exact composition has been the source of some debate (reviewed in [118]). Many proteins have been described as being more or less tightly associated with nucleoids, suggesting a core with mtDNA and TFAM at its centre and rings of components rippling out in ever more distant circles [119]. Proteins involved in mitoribosome biogenesis or mt-rRNA modification have been reported to interact with (inner circle) or be found close to (more distant circles) nucleoids [120–123]. A number of other post-transcriptional processes have also been associated with submitochondrial particles that are found close to the nucleoid [124]. Transcribed polycistronic mt-RNA units have to be processed and matured to generate functional mt RNA species. A number of the RNA modifying enzymes necessary for these processes have been located to areas referred to as RNA processing granules and these have also been found to be close to the nucleoid [123,125]. What were not originally defined as part of the core complex were mitoribosomal proteins, although crude mass spectrometry data on immunoprecipitated mitoribosomes did report the association of various nucleoid-associated proteins [116]. Recent careful analysis, however, indicates that a subset of MRPs associates with nucleoids in a transcription dependent manner [124]. This is consistent with the coupling of mt-RNA with MRPs immediately following transcription. Together these data suggest that there is a tightly coordinated ‘conveyer belt/assembly line’ starting with mtDNA transcription that generates the nascent mt-rRNA transcript and then by spatial positioning of components, regulates the assembly of fully modified functional 55S particles onto which matured mt-mRNAs are loaded. We are, however, still far from a complete understanding of how these processes are spatially and temporally coordinated.

## 11 Why can’t we answer some of these questions using reporters or an *in vitro* translation system?

Reporter constructs are widely used to test various aspects of gene expression. Determining the consequences of mutations in open reading frames that might impact on efficiency of translational initiation or termination would be informative. Introducing changes to the sequence but not the predicted structures would also be useful to help identify the elements that are important in cleavage of sites in the polycistronic unit that are not at tRNA junctions. Unfortunately, neither the introduction of such reporter constructs into mitochondria, nor insertion of direct changes into mtDNA, is currently possible. As mentioned in the introduction, there is still no robust, reproducible system for transfection of mammalian mitochondria. The problems associated with this have been thoroughly reviewed and so will not be dealt here [9,10].

Cell free synthesis has been readily achieved for the eukaryotic and eubacterial system and wheat germ lysates are also commercially available. *Why should mitochondrial systems be so much more difficult?* Almost all of the initial attempts to establish *in vitro* mitochondrial translation systems started with a heterologous system often combining yeast or occasionally mouse mt-mRNAs, or simply homoribopolymers together with a mix of yeast and eubacterial components [126–128]. Short peptides could be synthesised from poly(U) or poly(UG) templates but only in the presence of eubacterial supplements, whilst programming with either poly(A) or poly(C) templates resulted in two orders of magnitude less product [128]. Other groups reported no nascent peptide production, or in some cases truncated or aggregated products [126,129]. Explanations proposed for these aborted syntheses included the change of UGA to a tryptophan codon, lack of recognition of the correct AUG initiator, or the necessity for organelle specific tRNAs [130–132]. The aggregation previously detected could occur either in the exit tunnel or as the peptide emerges from the exit site. It is easy to see how each of these scenarios could cause the ribosome to stall and thus generate only truncated species. Another difficulty is isolating translation competent mammalian mitoribosomes. As mentioned earlier, preparations of isolated mammalian mitoribosomes retain a deacylated P-site tRNA even after puromycin treatment and therefore could not be used. The alternative would be the daunting task of over-expression and purification of the 80+ components and constituents, including presumably modified, mt-rRNAs. This would be especially challenging, as there would be no guarantee that once purified and combined, these components would behave as the eubacterial preparations and dutifully self-assemble. All of the, sadly abortive, attempts so far point towards the need for an *in vitro* translation system to contain very specific mitochondrial factors from a homologous system. It seems highly probable that a successful system will also require the presence of a membrane into which the products can be co-translationally inserted to prevent aggregation, and the relevant chaperones. It may also prove that critical components have yet to be identified. There is no doubt that having such a system would greatly enhance our progress in understanding post-transcriptional gene expression in mammalian mitochondria. Unfortunately this *commodity* may remain elusive a while longer. Without it, even the most impressive high resolution structural analyses may only be giving a partial picture.

As the title indicates, there are still plenty of unanswered questions, far more than just those enumerated here. Unravelling the vagaries and intricacies of the mammalian mitochondrial translation system will keep the field busy for some time to come.

## Conflict of interest

There are no conflicts of interest.
